# Cerebello-Cortical Differences in Effective Connectivity of the Dominant and Non-dominant Hand during a Visuomotor Paradigm of Grip Force Control

**DOI:** 10.3389/fnhum.2017.00511

**Published:** 2017-10-25

**Authors:** Eric Moulton, Cécile Galléa, Claire Kemlin, Romain Valabregue, Marc A. Maier, Pavel Lindberg, Charlotte Rosso

**Affiliations:** ^1^Sorbonne Universits, UPMC Univ Paris 06, Inserm U1127, Centre National de la Recherche Scientifique UMR 7225, UM 75, ICM, Paris, France; ^2^Centre de Neuro-Imagerie de Recherche, CENIR, Paris, France; ^3^Université Paris Diderot, Sorbonne Paris Cité, Paris, France; ^4^FR3636, Centre National de la Recherche Scientifique, Université Paris Descartes, Sorbonne Paris Cité, Paris, France; ^5^INSERM U894, Université Paris Descartes, Sorbonne Paris Cité, Paris, France; ^6^AP-HP, Urgences Cérébro-Vasculaires, Hôpital Pitié-Salpłtrire, Paris, France

**Keywords:** dynamic causal modeling, fMRI, handedness, cerebellum, visuomotor

## Abstract

Structural and functional differences are known to exist within the cortical sensorimotor networks with respect to the dominant vs. non-dominant hand. Similarly, the cerebellum, a key structure in the sensorimotor network with its cerebello-cortical connections, has been reported to respond differently when using the dominant vs. non-dominant hand. Several groups have already investigated causal interactions during diverse motor paradigms using effective connectivity but few have studied the larger visuomotor network, including key structures such as the parietal cortex and the cerebellum, with both hands. Moreover, the effect of force level on such interactions is still unclear. We therefore sought to determine the hemispheric asymmetries in the cerebello-cortical sensorimotor network in right-handers at two force levels (5% and 10% maximum voluntary contraction) for both hands. Cerebello-cortical modulations were investigated in 28 healthy, right-handed volunteers by determining the effective connectivity during a visuomotor task at two force levels under fMRI. A network was built consisting of the left and right primary motor (M1), ventral premotor (PMv) and posterior parietal cortices (PPC), in addition to the supplementary motor area (SMA), and the ipsilateral cerebellum (Cer) to the hand performing the motor task. Task performance (precision of isometric grip force tracking) did not differ between hands, nor did task-related activations in the sensorimotor areas apart from the contralateral primary motor cortex. However, during visuomotor control of the non-dominant hand, connectivity analysis revealed causal modulations between (i) the ipsilateral cerebellum and SMA, and (ii) the ipsilatearl cerebellum and contralateral PPC, which was not the case when using the dominant hand. These cerebello-cortical modulations for the non-dominant hand were more present at the higher of the two force levels. We conclude that precision force generation executed with the non-dominant hand, compared to the dominant hand, may require enhanced cerebello-cortical interaction to ensure equivalent left-right task performance.

## 1. Introduction

Growing evidence suggests that there are hemispheric differences in the sensorimotor network in the healthy population at both the structural and functional level (Mattay et al., 1998; Westerhausen et al., 2007; Barber et al., 2012; Mutha et al., 2013). Dissociating the effects of laterality in the human motor system is therefore important, notably when studying patients with focal brain lesions. Many studies on post-stroke motor deficits consider the left and right hemisphere as being equivalent, pooling left and right-sided deficits (Grefkes et al., 2008b; Schaechter et al., 2009; Schulz et al., 2016), in particular when limited statistical power prevents studying stroke laterality as an explanatory factor. However, studies in various stroke populations have already shown differences in impairment based on whether the dominant or non-dominant hand was affected (Haaland et al., 2004; Harris, 2006; Kemlin et al., 2016). In order to fully understand the dynamics and outcome of upper limb motor recovery following focal brain damage, it is crucial to further elucidate the inherent differences in the motor network that concern the dominant or non-dominant hand as a function of force level in healthy subjects. In this study, we were particularly interested in hemispheric differences involving the cerebellum.

In terms of sensorimotor control, the cerebellum is crucial for motor initiation, motor adaptation, and error correction: it receives, integrates, and conveys information to the parietal and premotor cortices (Ramnani, 2012; Sokolov et al., 2017). In addition, the ipsilateral cerebellum has been shown to be more active for movements of the non-dominant than the dominant hand (Jäncke et al., 1999). We predicted that hand-dominance should therefore be reflected in the functional interactions (here, effective connectivity) between the cerebellum and the cerebral cortex. We investigated these functional interactions using Dynamic Causal Modeling (DCM). DCM is a technique which allows to quantify the causal interactions between distant regions in the brain and therefore constitutes a powerful method of investigating network dynamics noninvasively. In DCM, an a priori network structure is established by choosing regions of interest (ROIs) known to belong to a given network. Several groups have already investigated causal interactions during diverse motor paradigms using effective connectivity (Grefkes et al., 2008a,b; Bönstrup et al., 2016). Of the small number of studies which have investigated causal interactions for both hands, there seems to be few differences at the cortical level (Pool et al., 2014). However, the cerebello-cortical interaction was generally not investigated in such studies, despite the fact that the cerebellum seems to play a differential role for motor execution with the dominant or non-dominant hand (Mattay et al., 1998). Furthermore, cerebellar modulations onto regions of the cortex likely reflect different aspects of sensorimotor integration (Doyon and Benali, 2005), which provide varying degrees of information as a function of force level (Proske and Gandevia, 2012).

To this end, we performed a more comprehensive analysis using DCM based on a visuomotor force-tracking task, taking into account bilateral parietal, motor, premotor and cerebellar regions of interest (ROIs) to construct a sensorimotor network model. The inclusion of these particular ROIs was critical for elucidating the underlying network dynamics as they are all highly involved in visuomotor paradigms (Coombes et al., 2010). We manipulated two factors: the hand used to perform the task and the force level. These two factors allowed us to not only observe differences between the dominant and non-dominant hand but also to study the role of the cerebellum in modulating the motor network depending on the fineness of somatosensory integration.

The main goal of this study was to determine the differences (if any) in the cerebello-cortical modulations for (1) the dominant vs. the non-dominant hand and (2) force level. To this purpose, we will first describe the causal interactions in the sensorimotor network for the dominant and non-dominant hand during a visuomotor force-tracking paradigm and then analyze (if any) the relationship between force level and causal interactions in the sensorimotor network for each hand. We predicted that (1) the main cortical interactions would constitute an extensive, bilateral network and be nearly identical for the dominant and non-dominant hand (Pool et al., 2014); however, (2) the unfamiliarity of executing our visuomotor task with the non-dominant hand (Mattay et al., 1998) would require additional cerebello-cortical modulations than for task execution with the dominant hand. (3) Finally, we hypothesized that different force levels would result in varying cerebellar modulations to regions of the cerebral cortex involved in sensorimotor integration (Doyon and Benali, 2005).

## 2. Methods

### 2.1. Subjects

Twenty-eight healthy volunteers (38.0 ± 14.6 years old; 16 males) were recruited using the following criteria: (1) no history of neurological or psychiatric disorders determined through an interview with a trained neurologist (Mini-Mental Status Examination > 27), (2) age older than 18 years, (3) no contra-indications for MRI, (4) right-handedness and (5) normal or corrected vision. Volunteers were evaluated with the Edinburgh Handedness Inventory (EHI) (Oldfield, 1971). In all subjects, maximum voluntary contraction (MVC) was measured with a dynamometer (MIE, Medical Research Ltd., http://www.mie-uk.com/pgripmyo/index.html): the average of three attempts was retained for each hand. The study was approved by the appropriate legal and ethical authority (CPP Ile de France VI—Pitié-Salpêtrière, Paris, France) and was carried out according to guidelines of the World Medical Association (Declaration of Helsinki). Written informed consent was obtained from all participants.

### 2.2. Functional paradigm

**Figure 1:**
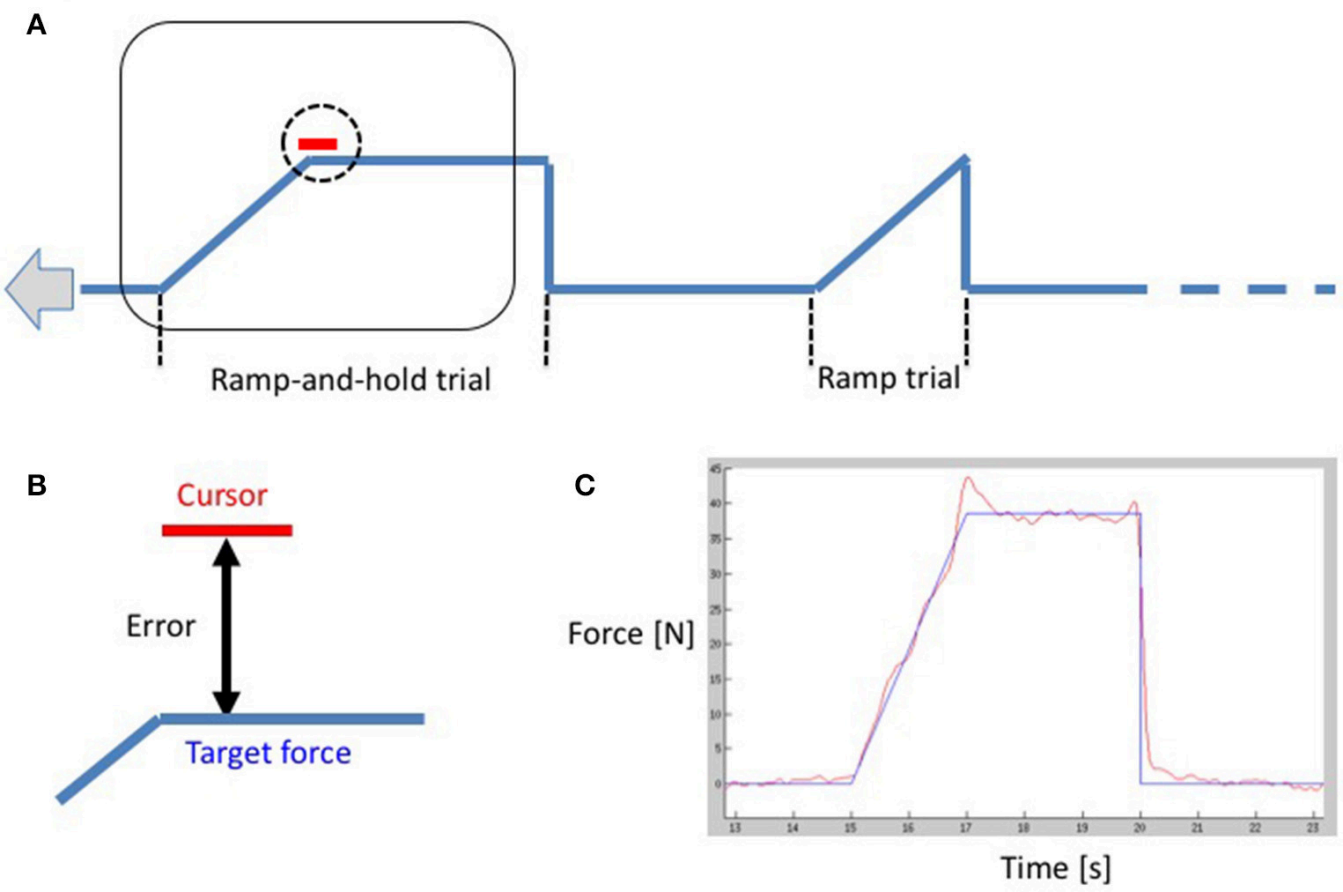
**(A)** A schematic drawing of the functional paradigm showing a ramp-and-hold trial and a ramp-trial. The curve moves from right to left with constant velocity, and the curser remains in the center of the screen as demonstrated by the rectangle. The subject is instructed to align the vertical cursor position (vertical position is proportional to the applied force) to the time-varying target force. **(B)** A close-up of the cursor. The (instantaneous) error is the deviation between the center of the cursor and the target force. **(C)** A subject's performance in red overlaid on the target force in blue during the task.

The functional paradigm was a mixed block-event related design: participants performed a visually guided, unilateral power grip ramp-and-hold force-tracking task (see Lindberg et al., 2009, Figure 1). Participants were instructed to follow a target force trajectory on a screen with a cursor controlled by a manipulandum held in the participants' (active) hand. By squeezing the manipulandum in a power grip, participants moved a cursor vertically on the screen in real-time and in proportion to the applied force. Increasing grip force produced an upward cursor movement, and decreasing, a downward cursor movement. The subjects followed the target trajectory consisting of successive trials. Each trial consisted either of (i) a linear ramp up and a subsequent instantaneous release (= “ramp event”), or (ii) a linear ramp up, a hold phase and a subsequent instantaneous release (= “ramp-and-hold event”). Ramp duration (2 s) and hold duration (3 s, if present) were kept constant. The type of trial was varied in order to keep subjects attentive. The pause between trials (zero grip force) varied randomly between 3 and 15 s (mean = 6.3 s, *SD* = 3.6 s). Zero force was equivalent to just holding/stabilizing the manipulandum in the hand. The task was performed twice with each hand individually. The target trajectory comprised seven blocks of three events: one ramp and two ramp-and-hold events. Two different target force levels were used: for the first run, the peak (hold) force was set at 10% MVC for four blocks and interleaved with three blocks at 5% MVC. The second run contained three blocks at 10% MVC interleaved with four blocks at 5% MVC. Subjects systematically began with the first run; however, the hand with which participants began was counter-balanced. A second manipulandum was placed in the inactive hand to control for eventual involuntary bilateral contractions. All subjects were trained until they felt comfortable with the task. For each trial, the root mean square (RMS) error between the target force trajectory and the subject's force trajectory was calculated and normalized by the target force level of the trial (Figure 1). We then averaged all errors within and across runs for 5% MVC and 10% MVC, respectively. We performed a repeated measures ANOVA with within-subject factors HAND and FORCE. *Post-hoc t*-tests were applied to determine the directionality of significant factors (*p* < 0.05) in order to establish if errors were attributable to handedness or force levels.

### 2.3. Functional magnetic resonance imaging

#### 2.3.1. Image acquisition

MRI data was obtained with a 3T scanner (Siemens, VERIO) with a 32-channel head coil. The MRI protocol included a sagittal T1-weighted MPRAGE image (TR = 2.3 s; TE = 4.18 s; flip angle = 9°; TI = 900 ms; voxel size 1×1×1 mm^3^; 176 slices), gradient-echo echo-planar functional images sensitive to BOLD contrast (TR = 2.1 s; TE = 25 ms; flip angle = 78°; voxel size 3×3×3.15 mm^3^; 110 volumes with anterior to posterior phase encoding + 4 volumes with posterior to anterior phase encoding, acquired in ascending order).

#### 2.3.2. Image preprocessing

The functional images were processed using SPM12 (http://www.fil.ion.ucl.ac.uk/spm) following standard procedures. The functional images were slice-time corrected and realigned to the first volume of the sequence to correct for head movements throughout the exercise. The functional images with posterior-anterior phase encoding were used to correct susceptibility distortions in the functional images using FSL's TOPUP (Andersson et al., 2003; Smith et al., 2004). The corrected volumes were normalized in MNI space by coregistering them onto the T1 and then applying the deformation field from the anatomical images. Finally, the functional images were smoothed using an isotropic 8-mm full width at half-maximum Gaussian kernel.

#### 2.3.3. First and second level analyses

A general linear model analysis was performed at the subject level using box-car conditions convoluted with the canonical hemodynamic response function in order to model average activation during all the trials. Head movement parameters were included as nuisance variables. Seven contrast images were computed for each subject and subsequently entered in a random-effects second-level analysis with subject age as a covariate: (1) right, dominant hand grasping, (2) left, non-dominant hand grasping, (3) a conjunction analysis of both hand conditions, (4) 10% MVC > 5% MVC for the right (dominant) hand, (5) 5% MVC > 10% MVC for the right hand, and (6) 10% MVC > 5% MVC for the left (non-dominant) hand, and (7) 5% MVC > 10% MVC for the left hand. Group-level statistical parametric map results are presented with a voxel-wise Family Wise Error (FWE) correction at *p*<0.05.

#### 2.3.4. ROI selection for DCM analysis

The visuomotor network consisted of 8 ROIs: left and right M1, ventral premotor cortex (PMv), posterior parietal cortex (PPC), a single left-right merged supplementary motor area (SMA), and lobule VI of the cerebellum (Cer) ipsilateral to the active hand. The inclusion of these particular ROIs was critical for elucidating the underlying network dynamics as they are all highly involved in visuomotor paradigms (Coombes et al., 2010; Mayhew et al., 2017). The cerebellar peak corresponds to lobule VI and was chosen primarily based on functional activations at the group-level and not on anatomical location. However, this location seems to be rather consistent with previous studies. In particular, a meta-analysis of the functional topography of the human cerebellum showed significant clusters in lobule VI (Stoodley and Schmahmann, 2009) for right-handed finger movement tasks, and functional MRI studies using similar visuomotor pardigms have reported large clusters of activity in lobule VI of the cerebellum (Vaillancourt, 2006; Coombes et al., 2010; Neely et al., 2013). We opted for a merged SMA primarily in order to reduce the number of ROIs (complexity) of our model, but also because our 8mm FWHM smoothing made the distinction between left and right SMA difficult. ROI coordinates were chosen based on the fMRI peaks from different group level activation maps (Supplementary Materials). Functional peaks for left M1 were found using runs with the right hand and vice-versa. For the remaining ROIs, peaks were found using the conjunction analysis across both hands, as these regions were activated bilaterally in visuomotor paradigms irrespective of the hand and as reported in previous studies (Coombes et al., 2010; Alahmadi et al., 2015; Bönstrup et al., 2016). Peak voxels were dilated by a 6 mm radius sphere using a mathematical morphology technique in order to constrain our ROIs to the local curvature of the sulci and gyri and to not encroach nearby functionally distinct areas. (Supplementary Materials)

#### 2.3.5. Dynamic causel modeling

**Figure 2:**
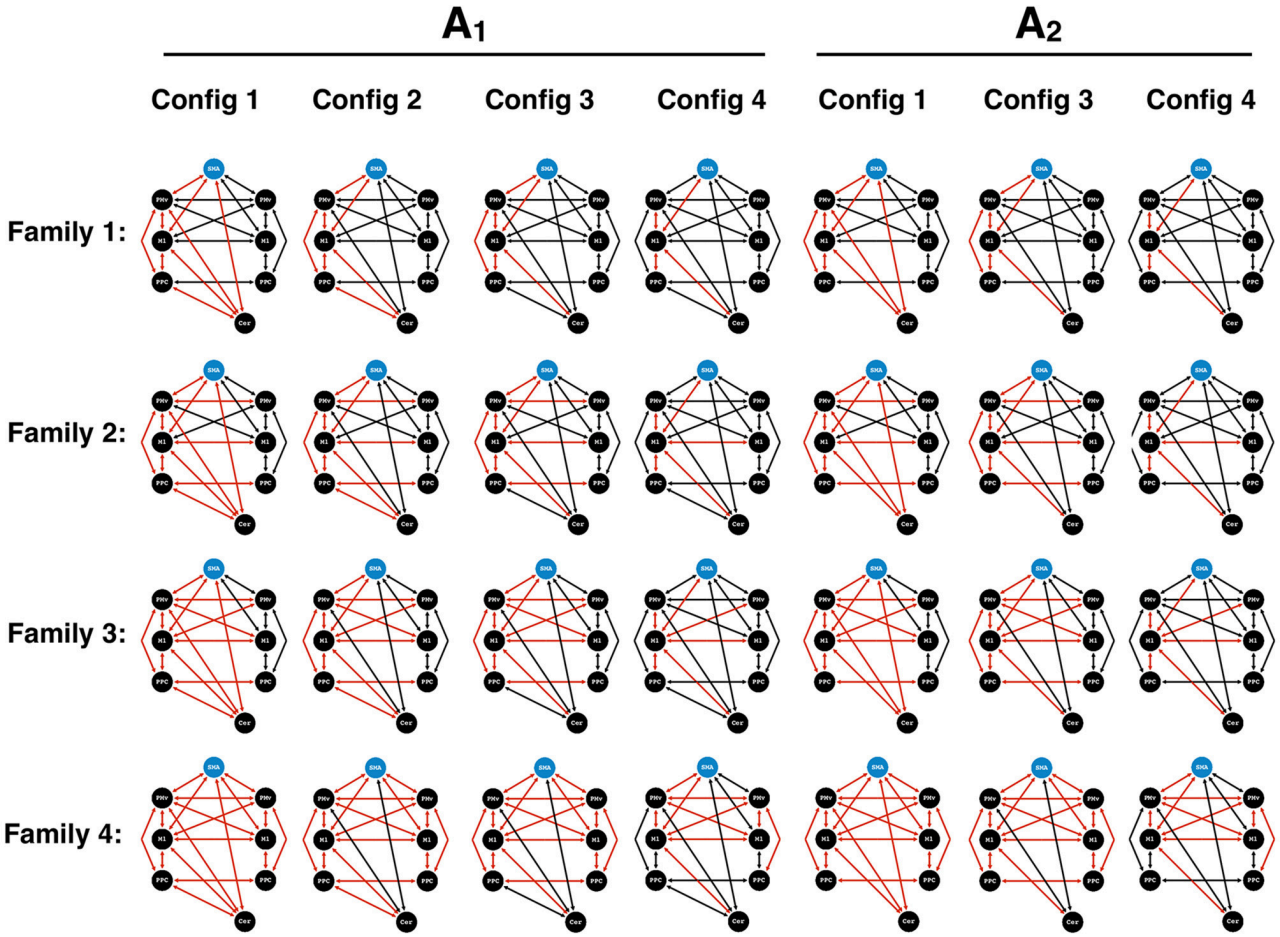
DCM models for Bayesian model comparison. Two endogenous connectivity matrices (A_1_ and A_2_) were proposed to test the presence of cerebello-parietal connections. Four families were created. Family 1 consisted of intrahemispheric modulations (red) contralateral to the hand performing the motor task. Family 2 contained additional transcallosal homotypic connections. Family 3 harbored heterotypic connections. Finally, Family 4 contained an extensive set of bilateral modulations. In each family, different configurations (Config) of the cerebello-cortical and parieto-premotor modulations were applied. Configuration 1 consisted of all possible cerebello-cortical. In configuration 2, the cerebello-premotor modulation was removed. In configuration 3, we proceeded to remove the cerebello-parietal modulation. Finally, in configuration 4, we removed the parieto-premotor modulation contralateral to the hand performing the motor task. SMA, supplementary motor area; PMv, ventral premotor cortex; M1, primary motor cortex; PPC, posterior parietal cortex; Cer, Cerebellum.

Dynamic Causal Modeling (Friston et al., 2003) was used to assess the effective connectivity between regions activated by the visuomotor paradigm. We extracted the first eigenvariate of the BOLD time-series, adjusted for confounds, from the 8 ROIs based on the group peak of the appropriate contrast maps and then adapted to subject-specific local maxima activations (*p* < 0.05, uncorrected). Four families of models (Penny et al., 2010) were constructed by systematically varying the modulations (B-matrix) for two sets of possible endogenous connectivity (A-matrix) based on anatomically and/or physiologically observed connections in humans and non-human primates involved in visuomotor processing (Wise et al., 1997; Boussaoud et al., 2005; Dancause et al., 2006, 2007; Stepniewska et al., 2006; Akkal et al., 2007; Koch et al., 2007; Feurra et al., 2011; Gharbawie et al., 2011; Schulz et al., 2015; Wang et al., 2015) (Figure 2). In the first A-matrix, all possible cerebellar connections to the contralateral hemisphere were present. In the second A-matrix, the cerebello-parietal connection was removed in order to test the necessity of these endogenous connections.

Based on the two A-matrices, the construction of each family began with a unique model containing all of the four cerebello-cortical modulations and served to test distinct patterns of intra and inter-hemispheric modulations of the motor, premotor, and parietal cortices.

Family 1, referred to as “unilateral”, contained modulations centered around contralateral M1, with no transcallosal modulations.
Family 2, termed “homotypic”, included homotypic transcallosal modulations.
Family 3, termed “heterotypic” included both homotypic and heterotypic modulations between motor, premotor, and parietal cortices.
Family 4, termed “bilateral”, consisted of bilateral modulatory connections.

In each family, different configurations of the cerebello-cortical and parieto-premotor modulations were applied. Configuration 1 contained all four of the cerebello-cortical modulations. In configuration 2, cerebellar modulations onto PMv and M1 were removed from configuration 1. In configuration 3, the cerebello-parietal modulations were removed from configuration 2. Finally, the last configuration saw a further removal of the parieto-premotor modulations contralateral to the hand performing the motor task. The possible combination of the standard models of each family and their variants resulted in a total of 28 different models. In each of these models, we placed the input (C-matrix) on the SMA for two reasons: (i) due to the role of the SMA in motor planning, similar to other DCM studies (Bönstrup et al., 2016), and (ii) since our visuomotor task allowed for motor preparation and planning, given that the target trace moved across the screen with constant velocity and from right to left with a time horizon of 5 s (the cursor in the middle the screen represented the actual time, and the screen showed 2.5 s of the upcoming target trace (to the right of the cursor), and 2.5 s of the target into the past (to the left). Identical models were used for the data of the 5and 10% MVC events, assuming that motor performance at these two force levels relied on the same underlying functional network. Finally, the same models constructed for the right hand were mirrored for the left hand; however, the ROI for the cerebellum was appropriately changed to the side ipsilateral to the active hand. A random effects Bayesian model selection (BMS) was performed to determine the most likely family given the observed fMRI data (Stephan et al., 2010) (chosen as the family with the highest exceedance probability). An additional BMS within the winning family was performed to determine the best model (i.e., a single model with the highest exceedance probability). Model parameters were extracted for each subject, and each connection was averaged across our group. One sample *t*-tests were conducted on the endogenous connections (A matrix) and both B matrices to investigate consistencies in the subjects' models (Penny et al., 2010; Stephan et al., 2010).

## 3. Results

### 3.1. Hand dominance and task (motor) performance

**Figure 3:**
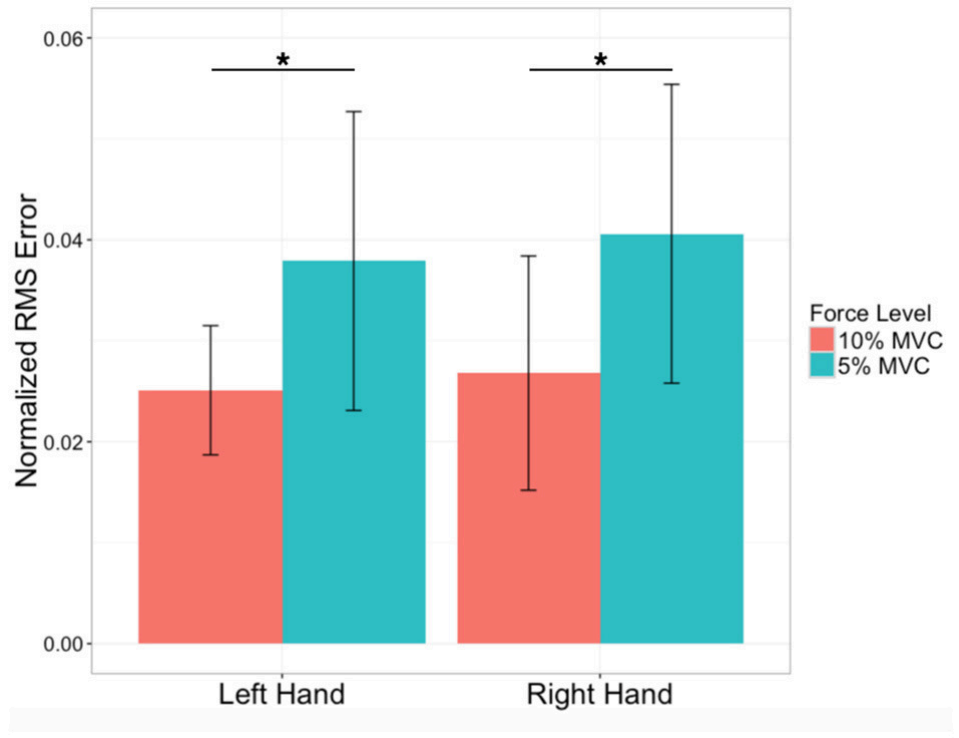
Performance errors. Average force-tracking errors expressed as Root mean square (RMS) errors normalized by force level for the left (non-dominant) and right (dominant) hand of N = 28 subjects. Variability indicated by ± 1 SD. ^*^Significant difference at *p* < 0.0001.

The mean and standard deviation for the handedness (EHI) scores were 0.87 ± 0.20 (N = 26). In terms of MVC grip strength, there was no significant difference between the right (307 ± 88N) and left (301 ± 82N) hand (*p* = 0.48). During the tracking task, RMS errors during the 5% and 10% MVC tasks were relatively low (Figure 3). There was no effect of left vs. right hand on RMS errors [*F*_(1, 27)_: 1.49, *p* = 0.2], but higher RMS errors were produced during 5% MVC events than 10% MVC events for both hands [*F*_(1, 27)_: 77.7, *p* < 0.0001, *post-hoc t*-tests, *p* < 0.0001].

### 3.2. Activation maps and force level differences

**Figure 4:**
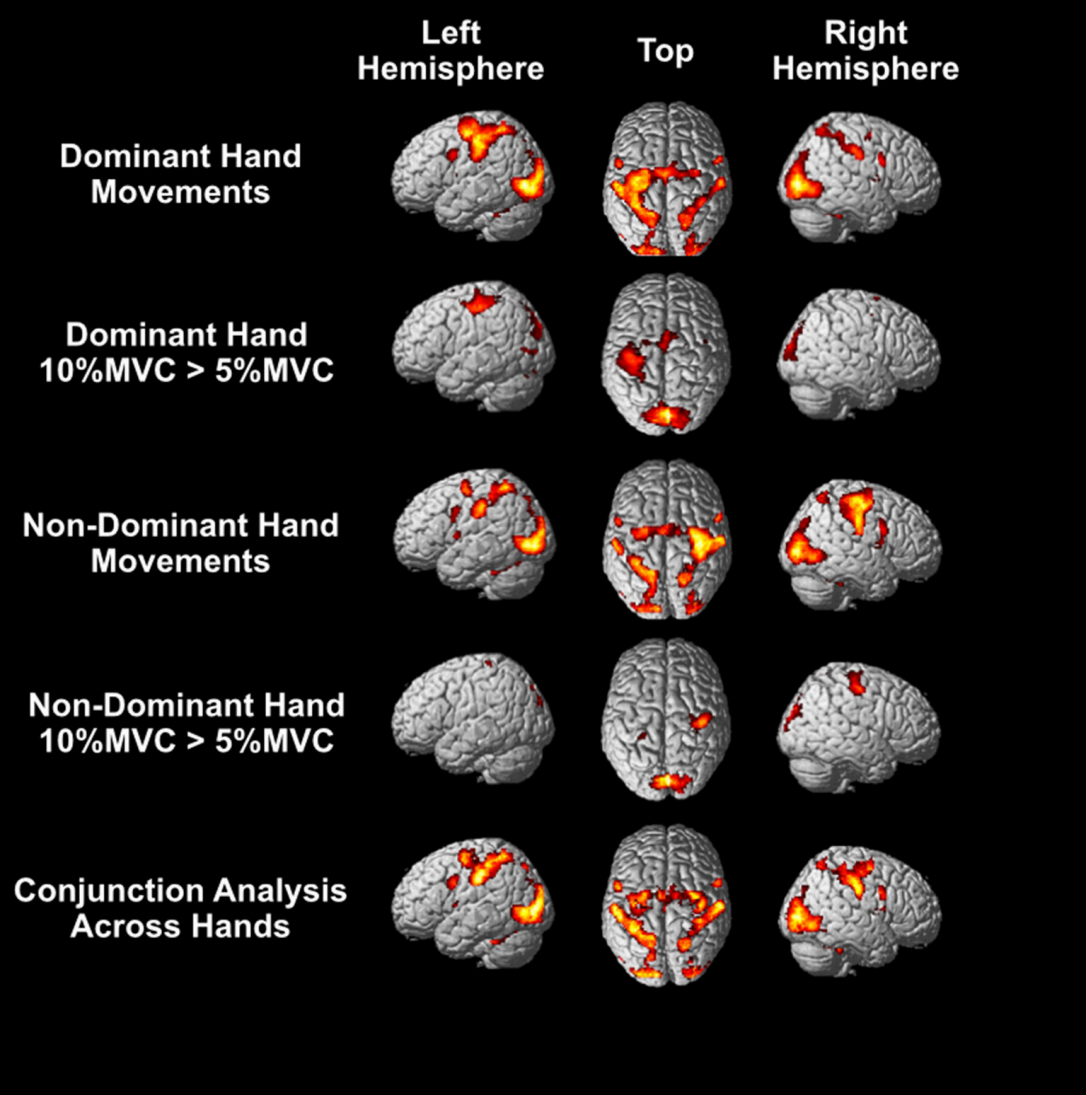
Group level activations. Top row: Activity for force-tracking with the right dominant hand. Second row: regions of higher BOLD response for 10% MVC than 5% MVC events for the right dominant hand. Third row: activity for force-tracking with the left non-dominant hand. Fourth row: regions of higher BOLD response for 10% MVC than 5% MVC events for the left non-dominant hand. Bottom row: the conjunction analysis for right and left hand grasping. All activations shown are FWE corrected voxel-wise at *p* < 0.05 (T > 5.78).

Bilateral activation was observed in many regions irrespective of the hand that performed the task: the SMA, dorsal and ventral premotor cortex, anterior intraparietal sulcus, posterior parietal cortex, inferior parietal lobule, extrastriate visual cortex (V3, V5), and cerebellum (Figure 4; see Supplementary Materials for peak coordinates). Unilateral activation was only observed for contralateral M1. For the right dominant hand, higher activity was observed in the left M1, SMA, and primary visual cortices for 10% MVC events vs. 5% MVC events (Figure 4), and for the left non-dominant hand, higher activity was observed for the right M1 and primary visual cortices. No area was more activated for the 5% MVC > 10% MVC contrast at the group level.

### 3.3. Motor system interactions for the dominant/non-dominant hands at two force levels

#### 3.3.1. Winning model for the dominant and non-dominant hand

**Figure 5:**
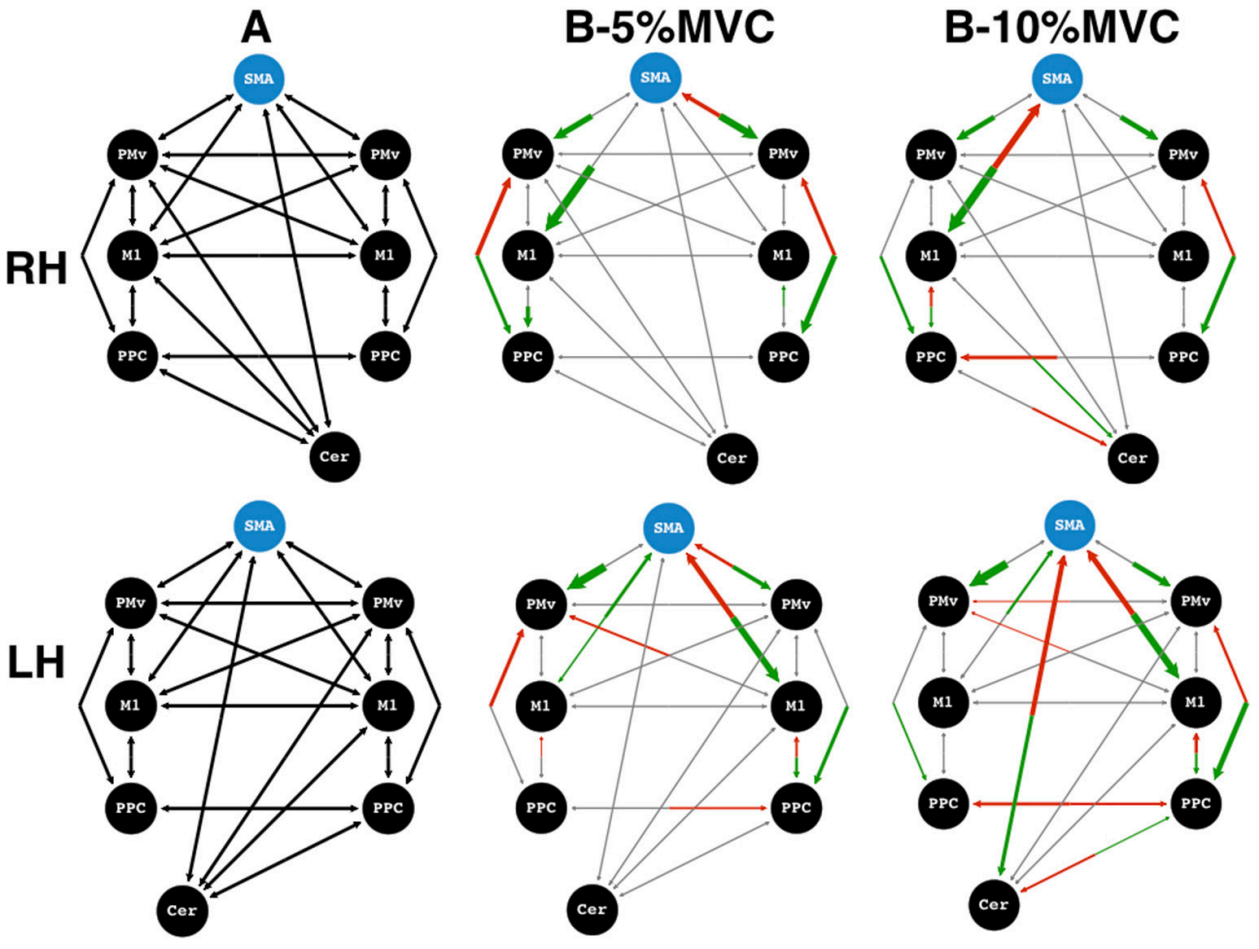
Winning models. Effective connectivity matrices for endogeneous connectivity (A-matrix) and coupled modulations (B-matrix) for force-tracking at 5 and 10% maximum voluntary contraction (MVC). Top row: dominant, right hand (RH), bottom row: non-dominant, left hand (LH). ROIs to the left of the supplementary motor area (SMA) region of interest are on the left hemisphere, and vice versa. Shown are the average connectivity with *p* < 0.05 (one-sample *t*-test). Green arrows represent significantly positive modulations, and red arrows represent significantly negative modulations. Gray arrows represent non-significant connections or non-present modulations and serve to show the underlying model structure. The blue circle indicates the input matrix (C-matrix). The thickness of the arrows for the B-matrices represents the strength of the connections. Connection strengths are normalized to the strongest connection of that matrix. All gray arrows are of the same thickness. SMA, supplementary motor area; PMv, ventral premotor cortex; M1, primary motor cortex; PPC, posterior parietal cortex; Cer, Cerebellum.

The Bayesian model selection between the four types of families revealed that the “bilateral” model (Family 4) explained the observed data better than the other 3 models for both the dominant and non-dominant hand (Figure 5, see Supplementary Materials for model comparison). However, subsequent comparisons within the “bilateral” family for the non-dominant and dominant hand revealed different winning models. (Figure 5, Supplementary Materials for parameter estimates and model comparison). While the winning endogenous matrix was the more fully connected of the two for both hands, the network difference between the dominant and non-dominant hand was contingent on cerebellar modulations: for the dominant hand, the winning model did not contain modulatory cerebello-premotor connections (i.e., SMA and PMv – configuration 2), whereas these connections were present for the network of the non-dominant hand (configuration 1) (Figure 5).

#### 3.3.2. Network comparison between dominant and non-dominant hands and as a function of force level

For trials at 10% MVC, the most striking difference between the network for the dominant and non-dominant hand was seen in the cerebello-cortical connectivity (Figure 5). At 10% MVC for the non-dominant hand, the cerebellum showed negative modulations onto the SMA, and a positive one onto the PPC. In return, the SMA had a positive and the PPC, a negative modulation onto the cerebellum ipsilateral to the active hand. For the right dominant hand, the same PPC to cerebellum modulation was present as for the left non-dominant hand. In addition, the contralateral M1 had a positive modulation onto cerebellar activity. However, in these cases the cerebellum did not modulate the ROIs of the cerebral cortex (M1, PMv, SMA or PPC).

Despite this difference, there were some network similarities between the dominant and the non-dominant hand at 10% MVC: (i) a positive influence from SMA to contralateral M1 with a negative feedback modulation, (ii) a positive contralateral M1 to PPC influence with a negative feedback modulation and (iii) a positive bilateral parieto-premotor influence for both hands. A different picture emerged for the 5% MVC events: cerebellar modulation onto cortical areas was absent for either hand. Interestingly, regardless of the active hand, there was an inhibitory feedback modulation from right PMv to SMA (i.e., the ipsilateral PMv for the dominant, and the contralateral PMv for the non-dominant hand). A further difference at the 5% MVC force level was the inhibitory modulation of M1 onto SMA for the left non-dominant hand, but absent for the right dominant hand. For the right hand, a bilateral negative influence of PPC onto PMv was present. For the left hand, however, this negative PPC-PMv modulation was present in the ipsilateral hemisphere. Finally, the more notable similarity between force modulatory connections (i.e., for both hands at both force levels) was a stronger positive influence from SMA to left and right PMv and to the M1 contralateral to the active hand.

## 4. Discussion

In the present study, we investigated the effective connectivity between eight regions of interest in the visuomotor (cortico-cortical and cerebello-cortical) network for the control of grip force of the dominant vs. non-dominant hand in right-handers. As expected, the most likely model for both hands contained bilateral modulations within and across both hemispheres, with several already reported cortico-cortical effective connections, such as strongly coupled positive modulations between SMA and bilateral PMv (Pool et al., 2014), and positive modulations from SMA to M1 contralateral to the active hand (Bönstrup et al., 2016). The novel observation concerns the cerebello-cortical network. The optimal models for the dominant and non-dominant hand differed in that causal cerebellum-SMA and cerebellum-PPC connections were present for the non-dominant hand, but not for the dominant hand. In particular, these modulations for the non-dominant hand arose when subjects performed the visuomotor task at 10% MVC, but not at 5% MVC.

### 4.1. Cerebello-cortical connectivity: Cer-SMA and Cer-PPC

Our study is the first to show a coupled cerebellar-SMA and cerebellar-PPC modulation (bidirectional, i.e., feed-forward and feedback) specifically during visuomotor force control with the non-dominant hand, but absent for the dominant hand. We interpret this finding as follows: (1) the particular cortical targets of the cerebellum being the SMA and the PPC suggest that these modulations sub-serve sensorimotor integration (visuomotor mapping, on-line adaptation, task monitoring) necessary for task completion, and (2) there is no evidence for this asymmetry in cerebello-cortical modulations to be related to task performance since force-tracking error did not differ when subjects used the dominant or non-dominant hand.

Why these cerebello-cortical modulations were, in our case, present at the higher (10% MVC) of the two tested force levels and only in the non-dominant hand may be explained by bringing up and combining two notions. First, this may be specific to the non-dominant hand, since control of the non-dominant hand (at similar performance levels compared to the dominant hand) may require more neuronal resources for sensory recalibration and motor adaptation (Ramnani, 2012; Sokolov et al., 2017). We assume that these extra neuronal resources put into play for the non-dominant hand are reflected in the observed cerebello-cortical modulations. It has been theorized that the coordinative role of the cerebellum in sensorimotor control consists in producing a forward model based on a copy of the motor command (generated in the SMA, as suggested by Haggard and Whitford, 2004). The forward model would allow for the prediction of the expected sensory consequences of that motor command, in combination with visual and proprioceptive sensory inputs from the parietal cortex (Blakemore and Sirigu, 2003; Sokolov et al., 2017). Tentatively, the significant bi-directional cerebellum-SMA and cerebellum-PPC modulations may represent this kind of increased processing for precise force-tracking of the non-dominant hand based on visual on-line error monitoring (Blakemore and Sirigu, 2003; Tseng et al., 2007). The dominant hand having more dexterous motor abilities, its use would not be challenging enough to call upon the engagement of cerebello-cortical network.

Second, why these cerebello-cortical modulations were, in our case, present at the higher (10% MVC) but not at the lower of the two tested force levels may be explained by another, complementary notion. The reliability of sensory modalities (in our task, vision and proprioception) used in force control may vary as a function of force level (Vaillancourt, 2003; Noble et al., 2013), and it is known that subjects rely in general on the more reliable modality, if two or more are available as was the case in our setup (Ronsse et al., 2009). Proprioceptive feedback, including its use in sense of effort signaling, is known to be more reliable at higher than at lower target forces (Proske and Gandevia, 2012). The region-of-interest in the PPC is near the caudal intraparietal sulcus: this area primarily integrates visual information into somatosensory representations of 3D space (Hadjidimitrakis et al., 2015; Piserchia et al., 2017), and together with the cerebellum ensures efficient performance of skilled hand movements (Blakemore et al., 2001). Therefore, enhanced proprioceptive feedback at 10% MVC may thus favorably bias the emergence of the Cer-SMA and Cer-PPC modulation, thought to represent sense of effort processing.

Altogether, we determined the effect of hand dominance and force level on the neural network responsible for the production of grip force. This results in differential force dependent patterns of connectivity between the dominant and non-dominant hand conditions. In particular, cerebello-parieto-premotor network would contribute to control the amplitude of grip force scaling by building and/or storing internal models to optimize motor skills with lesser amount of use-dependent practice.

### 4.2. Specific cortico-cortical motor connectivity (1): SMA-PMv

A positive coupled modulation from SMA to bilateral PMv was common to all four of our models. The SMA is known to harbor dense anatomical intra and inter-hemispheric connections with the ventral premotor cortex in non-human primates (Luppino et al., 1993; Dancause et al., 2006, 2007). This connection has been found in many motor-based DCM studies (Rehme et al., 2011; Pool et al., 2014; Schulz et al., 2016). Many different functional roles have been attributed to the SMA, most prominently control of self-triggered and of bimanual movements, as well as learning of motor sequences and of stimuli-response associations (Nachev et al., 2008). The extensive inter-hemispheric connections of the SMA is thought to reflect its implication in bimanual coordination (Dancause et al., 2007). In contrast, the PMv is, among other functions, involved in planning and scaling of finger kinematics (Dafotakis et al., 2008). Thus, the roles of these two areas in visuomotor control of voluntary hand movements appear to be rather complementary but interdependent: SMA and PMv have been thought to subtend transformation of visuo-spatial motor coordinates, necessary for “fast learning” (Dayan and Cohen, 2011). In our study, the strength of the SMA-PMv modulation was among the strongest in each model. A consistent positive SMA-PMv coupling, though not as strong, was also found during simple repetitive hand opening and closing movements (Pool et al., 2014). The stronger coupled modulations in the present study may be related to higher task difficulty (i.e., requiring continuous, fine-grained grip force adjustments to a visual target) or the PMv's well-established role in grasping objects (Castiello, 2005). Bönstrup et al. (2016) also investigated effective connectivity of motor and pre-motor areas using fMRI and EEG with a grip force task: similarly, they found a clear SMA-PMv modulation, which, however, varied from ours (in not being strictly bilateral, and in terms of signs: negative (EEG) as well as positive (fMRI) modulation). These differences may be explained by the somewhat divergent behavioral paradigms and differences in the analysis. Presumably higher task-related sensorimotor constraints and attentional demands may have induced stronger modulations between these two regions in our case.

### 4.3. Specific cortico-cortical motor connectivity (2): SMA-M1

A second consistent finding between all of our models was a positive and strongly coupled SMA-M1 modulation—a common finding in many motor-based DCM studies (Rogers et al., 2004; Grefkes et al., 2008a,b; Rehme et al., 2011; Pool et al., 2014; Bönstrup et al., 2016; Schulz et al., 2016). This connectivity, present for simple and complex tasks alike, has been attributed to the respective role of the SMA (preparation) and M1 (execution) in voluntary upper limb movements (performed with either hand, Rogers et al. (2004). For example, EEG phase-locking between SMA and M1 was observed at movement onset for externally cued movements and was attributed to the interaction between movement planning and execution (Myers and Mackinnon, 2004). However, if the presence of an SMA-M1 modulation has repeatedly been shown, the sign of this connectivity varied according to studies (likely due to particular behavioral paradigms and DCM models): Bönstrup et al. (2016) and we found no evidence for a negative modulation between SMA and M1 ipsilateral to the active hand, but this was reported in Grefkes et al. (2008a) and Pool et al. (2014).

### 4.4. Specific cortico-cortical motor connectivity (3): PMv-PPC

Additionally, in the right hemisphere, there was a positive coupling from the PPC to the PMv which was present for the dominant and non-dominant hand and at both force levels. There are numerous studies supporting the hypothesis that visual signals are transferred into motor commands through neural activity between the parietal and premotor cortical network (Goodale and Milner, 1992; Caminiti et al., 1996; Johnson et al., 1996), which have the particular role of programming movements before initiation in the right hemisphere (Terao et al., 2005). Makin et al. (2007) showed that the posterior intraparietal sulcus responds to objects around the perihand space and the anterior parietal sulcus integrates additional sensory information from perihand space, similar to the PMv (Ehrsson et al., 2001). That our observed PPC-PMv interactions were fairly consistent in the right hemisphere regardless of the hand used may be attributed to the right hemisphere dominance in spatial processing and movement initiation (Jager and Postma, 2003; Terao et al., 2005).

### 4.5. Limitations

Our study presents some methodological limitations which affect the extent of our interpretations. First, merging the left and right SMA precluded the investigation of several issues: determining a possible dominant role of the left vs. right SMA (Rogers et al., 2004), disentangling the contributions of left and right SMA modulations onto the contralateral motor cortices, and studying local left-right SMA-SMA interactions. Furthermore, whether the cerebellum projects to the left or right SMA remained open, although there is evidence to suggest that the ipsilateral cerebellum exclusively modulates the contralateral SMA (Akkal et al., 2007).

In addition, we did not fully explore the different possibilities of inputs (C-matrix) to our network. Many studies applying DCM to motor paradigms have chosen different C-matrices (input), with little consensus. For example, Wang et al. (2011) and Pool et al. (2014) put the C-matrix on bilateral PMv and SMA; Chen et al. (2010, 2012) used solely the lateral premotor cortex; Boudrias et al. (2012) used contralateral M1, SMA, PMv, and the dorsal premotor cortex (PMd). Due to these diverse approaches, Bönstrup et al. (2016) used Bayesian Model Selection (BMS) to choose between families of models with different C-matrices, including one with inputs on bilateral SMAs. Their BMS showed that no particular type of input was better than any other, indicating that the choice of input in the motor system has little bearing on the results. It therefore seemed reasonable to use a combined SMA region for input. In addition to this methodological argument, the role of SMA activity subtending premovement activity is well established (Nachev et al., 2008; Nguyen et al., 2014).

Finally, we did not include left-handers in our analysis. Consequently, we cannot ascertain that the same observed hemispheric asymmetries would be seen in left-handers in the left hemisphere or if they would remain in the right-hemisphere.

## 5. Conclusions

We provide evidence of differential effective connectivity in the visuomotor system of right-handers when using the dominant (right) vs. the non-dominant (left) hand during a visuomotor grip force task. The network model consisted of M1, SMA, PMv, PPC and the cerebellum. Compared to the dominant hand, additional effective connectivity was present during use of the non-dominant hand. This concerned modulations between the cerebellum and the SMA, as well as between the cerebellum and the right posterior parietal cortex. This reflects most likely additional neuronal resources required for monitoring motor execution during multi-sensorial force control of the non-dominant hand. Our results are largely consistent with but add more specificity to the reported network formed by these structures, all of which are involved in the execution of visuomotor tasks with the hand.

## Author contributions

EM, CG, CK, RV, MM, PL, and CR have met these 4 criteria: (1) substantial contributions to the conception or design of the work, (2) the acquisition, analysis, or interpretation of data for the work, (3) drafting the work or revising it critically for important intellectual content, (4) final approval of the version to be published. Agreement to be accountable for all aspects of the work in ensuring that questions related to the accuracy or integrity of any part of the work are appropriately investigated and resolved.

### Conflict of interest statement

The authors declare that the research was conducted in the absence of any commercial or financial relationships that could be construed as a potential conflict of interest.

## Abbreviations

M1: primary motor cortex
PMv: ventral premotor cortex
PPC: posterior parietal cortex
SMA: supplementary motor area
Cer: cerebellum
DCM: dynamic causal modeling
MRI: magnetic resonance imaging
EHI: Edinburg Handedness Inventory
MVC: maximum voluntary contraction
MPRAGE: Magnetization Prepared Rapid Acquisition Gradient Echo
TR: repetition time
TE: echo time
TI: inversion time
BOLD: blood-oxygen-level dependent
RMS: root mean square
ANOVA: analysis of variance
ROI: region of interest
FWE: family-wise error
BMS: Bayesian model selection
EEG: electroencephalography.
