# Supplementary material for: Cerebello-Cortical Differences in Effective Connectivity of the Dominant and Non-dominant Hand during a Visuomotor Paradigm of Grip Force Control
Source: Front Hum Neurosci. 2017 Oct 25;11:511. doi: 10.3389/fnhum.2017.00511 (PMC5662901; doi:10.3389/fnhum.2017.00511)
Supplement: Supplementary file 2 [file DataSheet2.pdf]

Supplementary Material: Cerebello-cortical differences in effective connectivity of the dominant and non-dominant hand during a visuomotor paradigm of grip force control

1 GROUP COORDINATES FOR REGIONS OF INTEREST IN THE DYNAMIC CAUSAL MODELING (DCM) ANALYSIS)

ROI	Coordinates	T-values
M1 L	-34,-24,48	10.49
M1 R	34,-20,50	10.76
PMv L	-54,4,34	10.95
PMv R	58,8,28	9.11
SMA	2,-6,52	6.98
PPC L	-18,-60,54	7.43
PPC R	22,-60,56	9.10
Cer L	-20,-62,-22	8.55
Cer R	22,-58,-24	9.84

Table S1. Group coordinates for ROIs used in Dynamic Causal Modeling (DCM) analysis. All voxels were FWE-corrected at $p<0.05$. T-values for the specific contrasts are shown. Abbreviations: L = Left; R = Right. M1 = primary motor cortex; SMA = supplementary motor area; PMv = ventral premotor cortex; PPC = posterior parietal cortex; Cer = Cerebellum.

2 REGION OF INTEREST CONSTRUCTION FOR THE DCM ANALYSIS

Peak voxels were dilated using geodesic dilation by means of the Nibabel and Mamba packages for the Python coding language (http://nipy.org/nibabel/,http://www.mamba-image.org/). Geodesic dilation is a mathematical morphology technique, which dilates a connected component constrained by the geometry of a mask image. The mask image was formed by averaging the subjects' normalized SPM-generated gray matter probability maps and thresholding at 30% probability. This threshold was determined visually by two experimenters (EM, CR) in order to include the maximum amount of gray matter and minimum amount of white matter, while avoiding bridging adjacent sulci for the ROIs in the DCM model. In the case of the SMA, the gray matter mask was manually bridged across hemispheres in order to ensure an unhindered dilation of the peak.

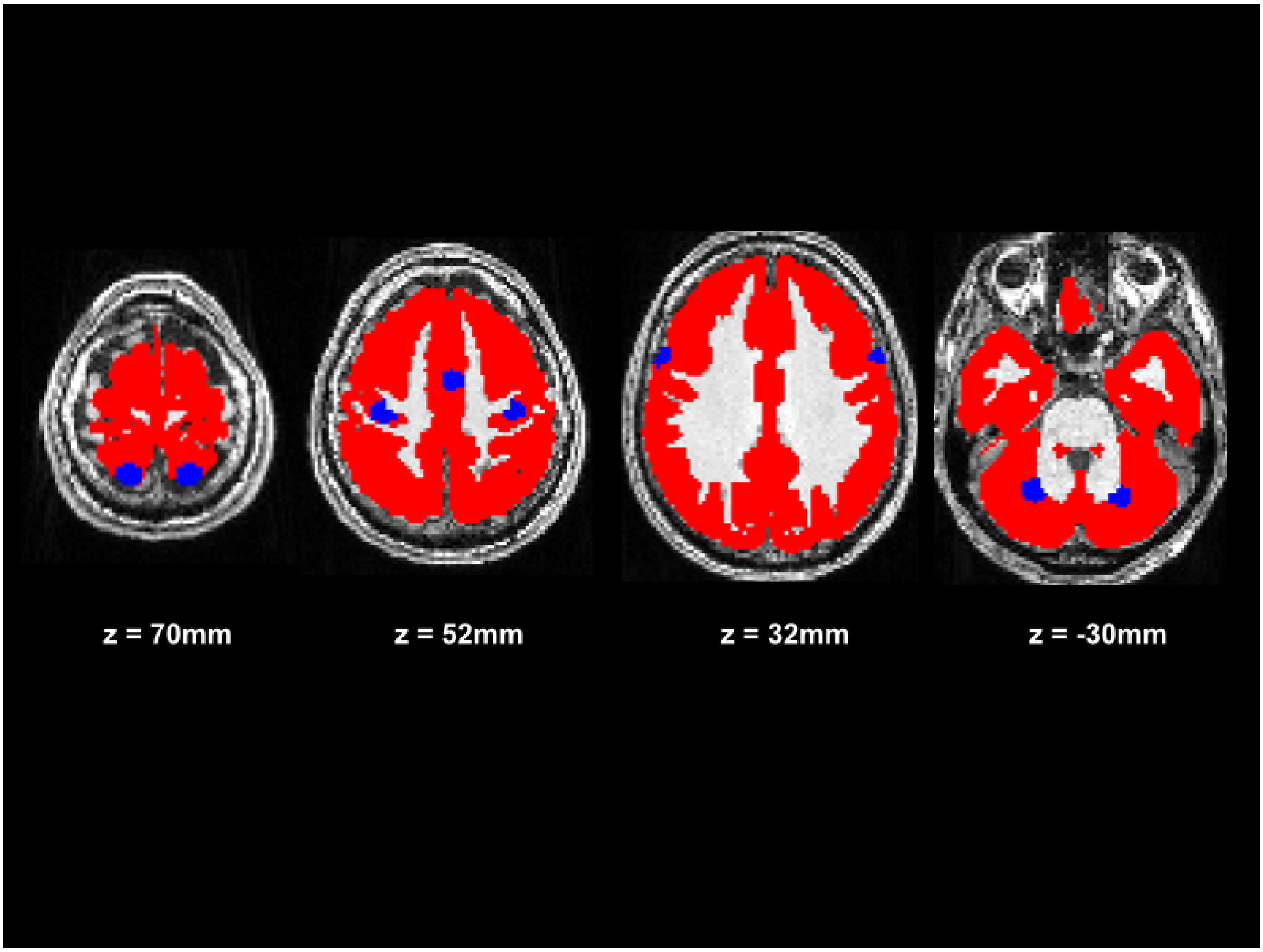

Figure S1: Geodesic dilation (Blue) of activation peaks within an averaged gray matter probability map (red) in a representative subject. ROIs for the DCM analysis were therefore restrained to areas of high probability of gray matter and avoided signal from adjacent gyri from the underlying whiter matter, or from non-brain tissue. Cuts are made at specific MNI coordinates to show the final ROIs for the posterior parietal cortex (z=70mm), primary motor cortex and the supplementary motor area (z=52mm), bilateral ventral premotor cortex (z=32mm), and the hand area of the cerebellum (z=-30mm).

3 BAYESIAN MODEL COMPARISONS

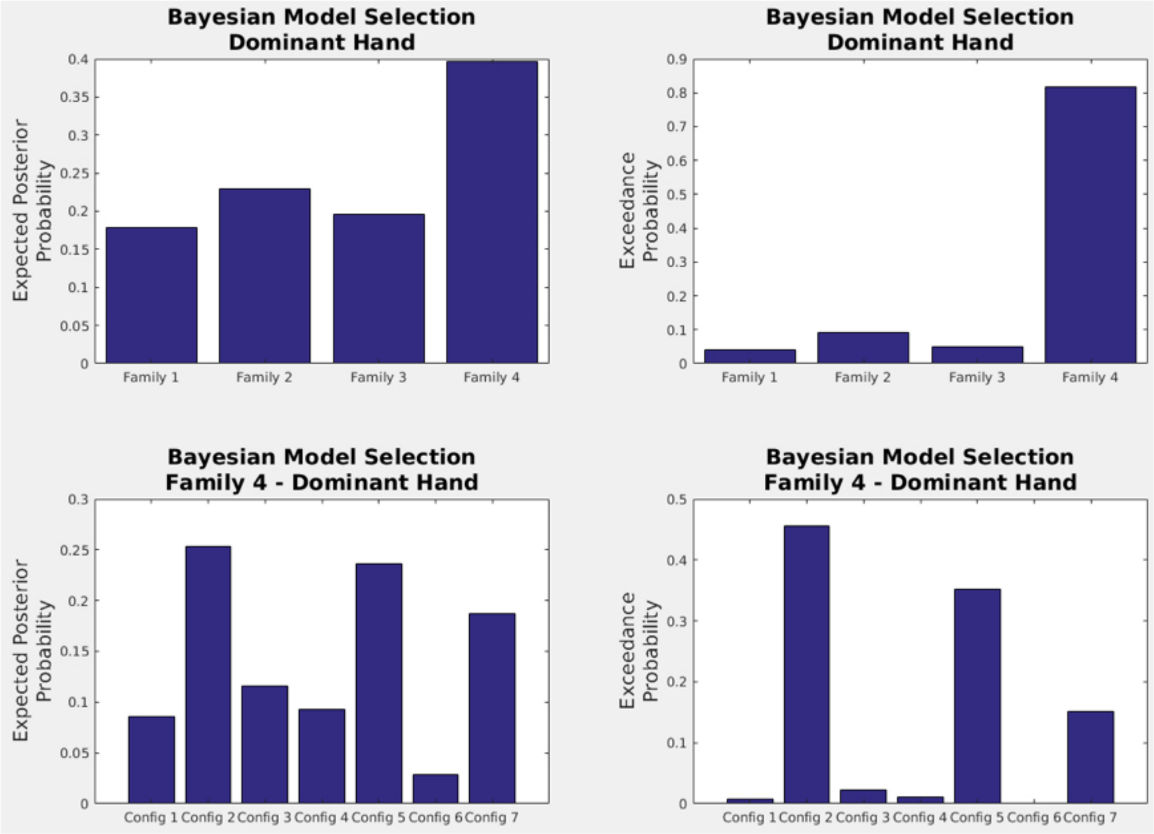

Figure S2: Bayesian Model Selection for for best Family and Model. Presented are the expected posterior probabilities and exceedance probabilities for comparisons of families and models for the dominant (right) hand.

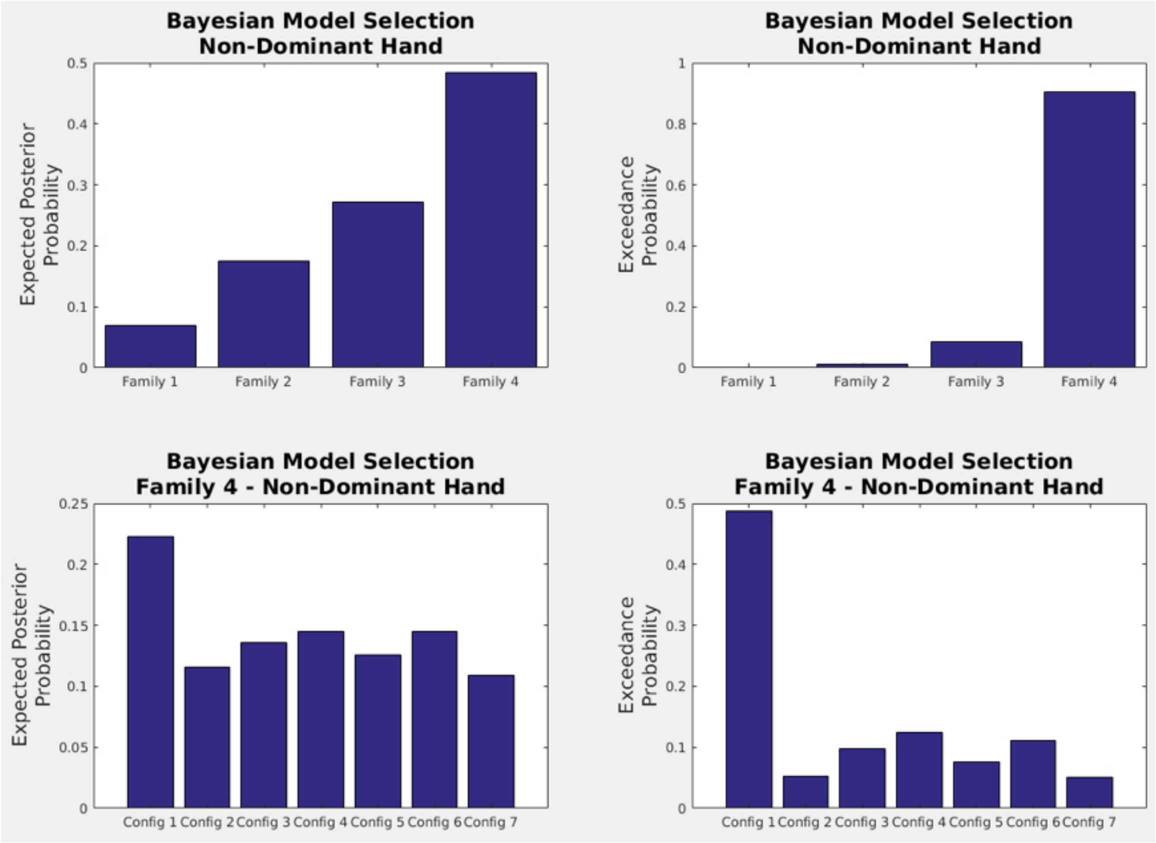

Figure S3: Bayesian Model Selection for for best Family and Model. Presented are the expected posterior probabilities and exceedance probabilities for comparisons of families and models for the non-dominant (left) hand.

4 ENDOGENOUS CONNECTIONS AND COUPLING STRENGTHS OF THE WINNING MODELS FOR THE DOMINANT AND NON-DOMINANT HAND

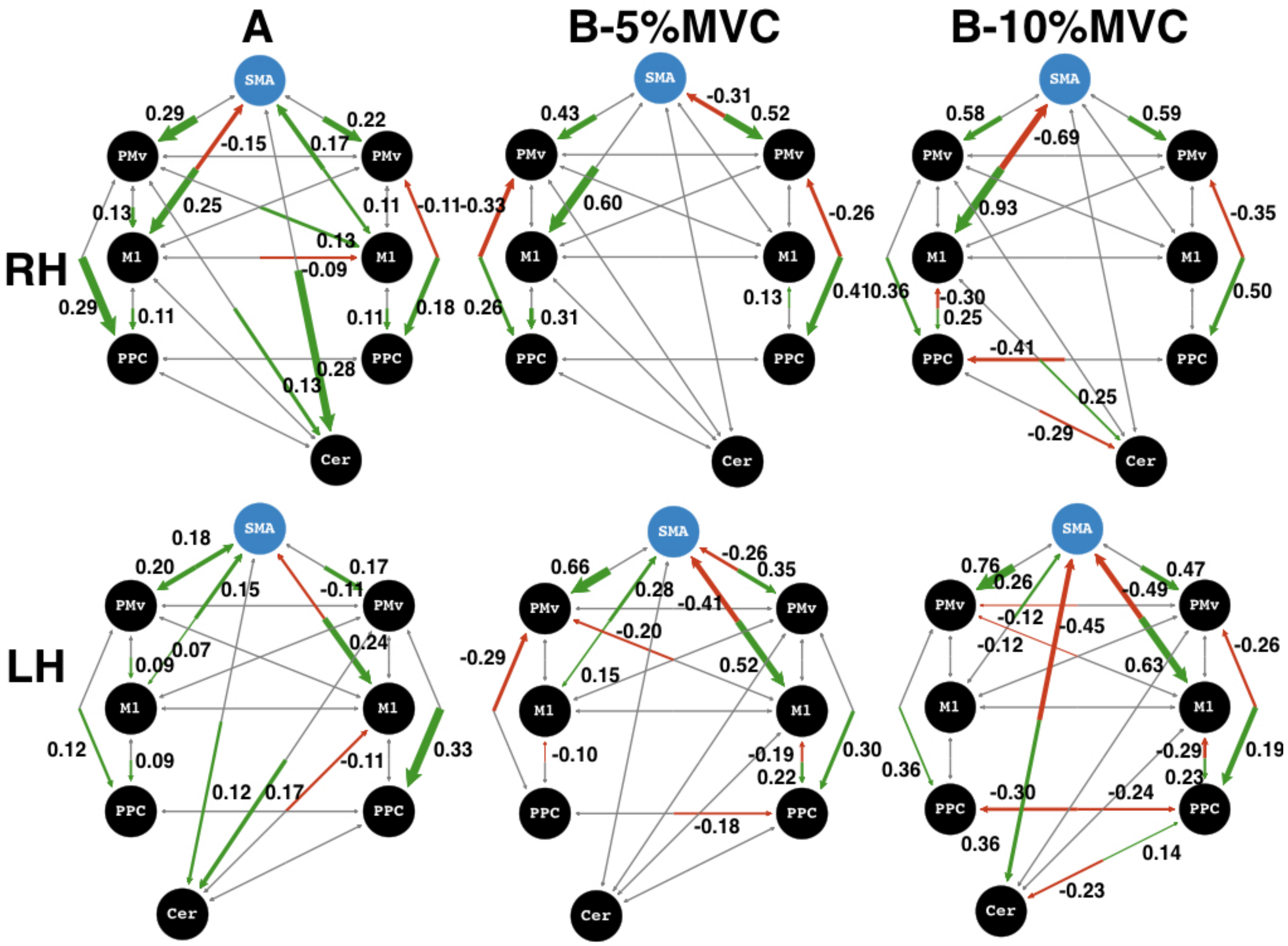

Figure S4: Winning Models. Effective connectivity matrices for endogeneous connectivity (A) and coupled modulations (B) for force-tracking at 5% and 10% maximum voluntary contraction (MVC). Top row: dominant, right hand (RH), bottom row: non-dominant, left hand (LH). ROIs to the left of the supplementary motor area (SMA) region of interest are on the left hemisphere, and vice versa. Shown are the average connectivity strengths which survived a one-sample t-test ($p<0.05$). Green arrows represent significantly positive modulations, and red arrows represent significantly negative modulations. Connection strength units are in Hz. Gray arrows represent non-significant connections or non-present modulations and serve to show the underlying model structure. The blue circle indicates the input matrix (C). The thickness of the arrows for the B-matrices represents the strength of the connections. Connection strengths are normalized to the strongest connection of that matrix. All gray arrows are of the same thickness. SMA = supplementary motor area; PMv = ventral premotor cortex; M1 = primary motor cortex; PPC = posterior parietal cortex; Cer = Cerebellum.
